# Supplementary material for: Analysis of inositol phosphate metabolism by capillary electrophoresis electrospray ionization mass spectrometry
Source: Nat Commun. 2020 Nov 27;11:6035. doi: 10.1038/s41467-020-19928-x (PMC7695695; doi:10.1038/s41467-020-19928-x)
Supplement: Supplementary file 3 — Reporting Summary [file 41467_2020_19928_MOESM3_ESM.pdf]

nature research

Corresponding author(s): Danye Qiu;Adolfo Saiardi;Henning J. Jessen

Last updated by author(s): Oct 25, 2020

Reporting Summary

Nature Research wishes to improve the reproducibility of the work that we publish. This form provides structure for consistency and transparency in reporting. For further information on Nature Research policies, see our Editorial Policies and the Editorial Policy Checklist.

Statistics

For all statistical analyses, confirm that the following items are present in the figure legend, table legend, main text, or Methods section.

n/a	Confirmed	
☐	☒	The exact sample size (n) for each experimental group/condition, given as a discrete number and unit of measurement
☐	☒	A statement on whether measurements were taken from distinct samples or whether the same sample was measured repeatedly
☒	☐	The statistical test(s) used AND whether they are one- or two-sided<br>*Only common tests should be described solely by name; describe more complex techniques in the Methods section.*
☒	☐	A description of all covariates tested
☒	☐	A description of any assumptions or corrections, such as tests of normality and adjustment for multiple comparisons
☐	☒	A full description of the statistical parameters including central tendency (e.g. means) or other basic estimates (e.g. regression coefficient) AND variation (e.g. standard deviation) or associated estimates of uncertainty (e.g. confidence intervals)
☒	☐	For null hypothesis testing, the test statistic (e.g. F, t, r) with confidence intervals, effect sizes, degrees of freedom and P value noted<br>*Give P values as exact values whenever suitable.*
☒	☐	For Bayesian analysis, information on the choice of priors and Markov chain Monte Carlo settings
☒	☐	For hierarchical and complex designs, identification of the appropriate level for tests and full reporting of outcomes
☒	☐	Estimates of effect sizes (e.g. Cohen's d, Pearson's r), indicating how they were calculated

Our web collection on statistics for biologists contains articles on many of the points above.

Software and code

Policy information about availability of computer code

Data collection	Agilent MassHunter Workstation Acquisition for Q-TOF B.04.00, Agilent OpenLAB CDS Chemstation 2.3.53
Data analysis	Agilent MassHunter Qualitative Analysis B.07.00, GraphPad Prism v.8.4.2

For manuscripts utilizing custom algorithms or software that are central to the research but not yet described in published literature, software must be made available to editors and reviewers. We strongly encourage code deposition in a community repository (e.g. GitHub). See the Nature Research guidelines for submitting code & software for further information.

Data

Policy information about availability of data

All manuscripts must include a data availability statement. This statement should provide the following information, where applicable:
- Accession codes, unique identifiers, or web links for publicly available datasets
- A list of figures that have associated raw data
- A description of any restrictions on data availability

Source data for generating figures are provided with this paper, as well as uncropped versions of gels and sequencing data. The raw mass spectrometric data files and all other relevant data supporting the findings of this study are available from the corresponding authors upon reasonable request.

Field-specific reporting

Life sciences study design

All studies must disclose on these points even when the disclosure is negative.

Sample size	The study focused on method development and triplicate and rarely duplicates for biological samples were chosen. Since some results are corresponding to earlier findings using other methods, more repeats could not bring significant improvements to the precision.
Data exclusions	For biological samples, no data are excluded, except one in Fig. 6g. We excluded this, since the extraction was unsuccessful.<br>CE-MS runs interrupted owing to technical problems are excluded, but it happened rarely.
Replication	The capillary was replaced once every two or three months. The described method is reproducible for each new capillary. From October 2019, our lab uses this method to analyse InsP species. At our request, the Agilent test lab also reproduced the analysis of InsP6 with similar results.
Randomization	Not applicable to this study, as samples were not assigned to experimental groups.
Blinding	Not applicable to this study, as there was no experimental group allocation in data collection and analysis.

Reporting for specific materials, systems and methods

We require information from authors about some types of materials, experimental systems and methods used in many studies. Here, indicate whether each material, system or method listed is relevant to your study. If you are not sure if a list item applies to your research, read the appropriate section before selecting a response.

Materials & experimental systems

n/a	Involved in the study	
☐	☒	Antibodies
☐	☒	Eukaryotic cell lines
☒	☐	Palaeontology and archaeology
☐	☒	Animals and other organisms
☒	☐	Human research participants
☒	☐	Clinical data
☒	☐	Dual use research of concern

Methods

n/a	Involved in the study	
☒	☐	ChIP-seq
☒	☐	Flow cytometry
☒	☐	MRI-based neuroimaging

Antibodies

Antibodies used	Santa Cruz Biotechnology ISYNA1 Antibody (C-9) mouse monoclonal IgG2b, catalogue number: sc-271830.<br><br>Santa Cruz Biotechnology Actin Antibody (I-19) goat polyclonal IgG: catalogue number: sc-1616, lot B0211<br><br>Sigma Aldrich Anti-Goat IgG–Peroxidase conjugated catalogue number: A5420<br><br>Sigma Aldrich Anti-Mouse IgG–Peroxidase conjugated catalogue number: A9044
Validation	The ISYNA1 antibody was validated using CRISPR generate (confirmed by sequencing) ISYNA1-/- clonal cell lines.<br><br>The anti Actin antibody is a widely used control antibody for western blot of human cell lines and used for example in PMID: 29239139; 27008544. This Actin antibody is cited >1640s see Santa Cruz website for the full citations list.

Eukaryotic cell lines

Policy information about cell lines

Cell line source(s)	HCT116 were acquired from ATCC, from witch both HCT116NIH and HCT116UCL originate (see validation). The other cell lines (HeLa, HT29, HEK293T, PC3, MCF7) were gifts from departmental colleagues and originally acquired from ATCC.
Authentication	The wild type HCT116UCL and HCT116NIH were validated by tandem DNA repeat sequences (STRs) using the ATCC cells profiling service as described (PMID: 27788189). HCT116UCLIP6K1,2-/- were characterized in (PMID: 31186349) HCT116NIHPPIP5K-/- were described in (PMID: 29078269). The other cell lines used in the manuscript where not authenticated.
Mycoplasma contamination	We assess mycoplasma contamination twice a year. The cell lines used tested negative for mycoplasma contamination.

Commonly misidentified lines (See ICLAC register)	No commonly misidentified cell lines were used in the study.

Animals and other organisms

Policy information about studies involving animals; ARRIVE guidelines recommended for reporting animal research

Laboratory animals	Mouse tissues, a gift from a departmental colleague, were dissected from 3 months old C57BL/6J female previously scarified to collect embryos. Mice were housed in a controlled environment with standard 12:12 light-dark cycle, temperature 19–22 °C, and humidity of 40-55%. All procedures relating to animal care and treatment conformed to the Institutional Animal Care and Use Committee at UCL.
Wild animals	This study does not involve wild animals
Field-collected samples	This study does not involve samples collected from the field.
Ethics oversight	No ethical approval was required since no mice were specifically scarified for this study.

Note that full information on the approval of the study protocol must also be provided in the manuscript.
